# Supplementary material for: Dehydroascorbic Acid Affects the Stability of Catechins by Forming Conjunctions
Source: Molecules. 2020 Sep 7;25(18):4076. doi: 10.3390/molecules25184076 (PMC7570458; doi:10.3390/molecules25184076)
Supplement: Supplementary file 1 [file molecules-25-04076-s001.pdf]

Table 1 Abbreviations and full name of substances

Abbreviation	Full Name
AA	Ascorbic acid
DHAA	Dehydroascorbic acid
EGCG	(-)-Epigallocatechin gallate
EC	(-)-Epicatechin
ECG	(-)-Epicatechin gallate
EGC	(-)-Epigallocatechin
GA	Gallic acid
GCG	Gallocatechin gallate
DKG	2,3-diketogulonic acid
TFs	Theaflavins
TSs	Theasinensins
GTC	Green tea catechins
TP	Tea polyphenols
